# Production, Structural Characterization, and In Vitro Assessment of the Prebiotic Potential of Butyl-Fructooligosaccharides

**DOI:** 10.3390/ijms21020445

**Published:** 2020-01-10

**Authors:** Sini Kang, Hyun Ju You, Yeong-Geun Lee, Yunju Jeong, Tony V. Johnston, Nam-In Baek, Seockmo Ku, Geun Eog Ji

**Affiliations:** 1 Department of Food and Nutrition, Research Institute of Human Ecology, Seoul National University, Seoul 08826, Korea; kangsini@snu.ac.kr (S.K.); tanklov0@snu.ac.kr (Y.J.); 2 Institute of Health and Environment, Graduate School of Public Health, Seoul National University, Seoul 08826, Korea; dhlover1@snu.ac.kr; 3 Graduate School of Biotechnology and Department of Oriental Medicinal Biotechnology, Kyung Hee University, Yongin 17104, Korea; lyg629@nate.com (Y.-G.L.); nibaek@khu.ac.kr (N.-I.B.); 4 Fermentation Science Program, School of Agriculture, College of Basic and Applied Sciences, Middle Tennessee State University, Murfreesboro, TN 37132, USA; tony.johnston@mtsu.edu; 5 Research Center, BIFIDO Co., Ltd., Hongcheon 25117, Korea

**Keywords:** prebiotics, butyl-fructooligosaccharides, short chain fatty acids, butyrate, structural analysis, NMR, gut microbiota, 16S rRNA metagenomic analysis

## Abstract

Short-chain fatty acids (SCFAs), especially butyrate, produced in mammalian intestinal tracts via fermentation of dietary fiber, are known biofunctional compounds in humans. However, the variability of fermentable fiber consumed on a daily basis and the diversity of gut microbiota within individuals often limits the production of short-chain fatty acids in the human gut. In this study, we attempted to enhance the butyrate levels in human fecal samples by utilizing butyl-fructooligosaccharides (B-FOS) as a novel prebiotic substance. Two major types of B-FOS (GF3-1B and GF3-2B), composed of short-chain fructooligosaccharides (FOS) bound to one or two butyric groups by ester bonds, were synthesized. Qualitative analysis of these B-FOS using Fourier transform infrared (FT-IR) spectroscopy, matrix-assisted laser desorption/ionization time-of-flight mass spectrometry (MALDI-TOF-MS), nuclear magnetic resonance (NMR) and low-resolution fast-atom bombardment mass spectra (LR-FAB-MS), showed that the chemical structure of GF3-1B and GF3-2B were [*O*-(1-buty-*β*-D-fru-(2→1)-*O*-*β*-D-fru-(2→1)-*O*-*β*-D-fru-*O*-α-D-glu] and [*O*-(1-buty)-*β*-D-fru-(2→1)-*O*-*β*-D-fru-(2→1)-*O*-(4-buty)-*β*-D-fru-*O*-α-D-glu], respectively. The ratio of these two compounds was approximately 5:3. To verify their biofunctionality as prebiotic oligosaccharides, proliferation and survival patterns of human fecal microbiota were examined in vitro via 16S rRNA metagenomics analysis compared to a positive FOS control and a negative control without a carbon source. B-FOS treatment showed different enrichment patterns on the fecal microbiota community during fermentation, and especially stimulated the growth of major butyrate producing bacterial consortia and modulated specific butyrate producing pathways with significantly enhanced butyrate levels. Furthermore, the relative abundance of *Fusobacterium* and ammonia production with related metabolic genes were greatly reduced with B-FOS and FOS treatment compared to the control group. These findings indicate that B-FOS differentially promotes butyrate production through the enhancement of butyrate-producing bacteria and their metabolic genes, and can be applied as a novel prebiotic compound in vivo.

## 1. Introduction

Short-chain fatty acids (SCFAs), also known as volatile fatty acids (e.g., formic, acetic, propionic, butyric, isobutyric, valeric and isovaleric acids), are composed of fewer than six carbons with straight or branched-chain structures and are mainly synthesized by naturally occurring gut microbiota in the intestinal lumen as a result of dietary fiber catabolism [1]. The most abundant SCFAs (≥95%) produced via carbohydrate metabolism of intestinal microbiota are acetate (C2), propionate (C3), and butyrate (C4). Among the various SCFAs, butyrate has received significant attention from scholars over the past few years, mostly due to its role as an energy source and range of health benefits. Specifically, butyrate plays a crucial role in colonic epithelial homeostasis by (i) improving proliferation of normal colonic epithelial cells, (ii) prompting sodium, potassium and water absorption, (iii) inhibiting colon inflammation and oxidative stress, (iv) maintaining the colonic epithelial barrier function, and (v) being the main fuel for colonocytes [2]. The anti-inflammatory activities and strengthening of epithelial barrier integrity make butyrate an ideal therapeutic treatment against gastrointestinal inflammation, such as inflammatory bowel disease (IBD) [3]. In addition, it has been reported that butyrate metabolism is impaired in the intestinal mucosa of IBD patients, resulting in butyrate reduction [4].

Despite the diverse biological functions of butyrate, there are potential limitations to the production of butyrate if the human diet is relied upon for the treatment or prevention of disease. Low fermentable fiber consumption and the diversity of gut microbiota within individuals often limits the production of butyrate in human intestines. Direct oral administration of butyrate is possible, but is not desirable due to its rancid taste and undesirable aroma. In addition, butyrate may also quickly be absorbed before reaching the large intestine, rendering oral administration ineffective. Although butyrate administration via rectal enema is utilized as a clinical therapy for colonic and rectal diseases, it is cumbersome and has only minor effects on mild inflammation [5,6,7].

To overcome these technical and adaptive problems, one strategy suggested by researchers for increasing butyrate levels in the intestinal tract is to orally supplement butyrate via coated pellets or tablets, using a pH-dependent coating or a hydroxyl propyl methylcellulose (HPMC) coating, to prevent early absorption before reaching the colon [8,9]. However, butyrate is possibly not liberated from tablets at the intended location due to differences in gut lumen pH and transit time amongst individuals [3]. An alternative approach is to develop butyrate-releasing derivatives, including butyrylated starch, butyryl-l-carnitine and N-(1-carbamoyl-2phenylethyl) butyramide [6,10,11]. Others have proposed the direct administration of colonizing butyrate-producing bacteria or the administration of prebiotic substances, which would affect the colonic butyrate-producing microbiome, ultimately resulting in the promotion of butyrate production [3]. The exhaustion of butyrate-producing bacteria is also associated with IBD [12]. Among the various prebiotic substances available, fructooligosaccharide (FOS) is a non-digestible oligosaccharide utilized as a nutraceutical supplement due to its bioactive properties [13]. The structure of FOS is an inulin-type oligosaccharides of D-fructose attached by β-(2→1) linkages that carry a D-glycosyl residue at the end of the chain [14]. Dietary FOSs are minimally hydrolyzed by digestive enzymes in the small intestine, which enables FOS to reach the large intestine without structural change [14,15]. Numerous studies have indicated that FOS is selectively consumed by probiotic bacteria (e.g., *Bifidobacteria*), favorably stimulates the growth of probiotic bacteria and further promotes well-balanced gut microbiota [16,17,18]. Recently, interest in prebiotics by food consumers has increased and numerous prebiotic products are being produced for the nutraceutical market. Producers are making efforts to produce products with differentiation and specificity in functionality to dominate the market [19].

In this study we propose and evaluate a new method of increasing intestinal butyrate levels by combining technologies mentioned earlier to generate biofunctional effects. We synthesized a novel prebiotic molecule, FOS linked to butyrate (B-FOS), as a potential prebiotic. The binding of FOS in B-FOS can be a strategy for oral delivery of butyrate to the distal colon, resulting in modification of the gut microbiota, which ultimately promotes colonic health. The chemical structure of B-FOS was identified in this study. To profile the impact of B-FOS on gut microbiota and butyrate production versus control and FOS groups, in vitro anaerobic fermentation was conducted in a broth mixed with human feces, which was intended to mimic the human intestinal environment. Changes in the microbial population over the course of fermentation were analyzed by 16S metagenomic techniques.

## 2. Results

### 2.1. Structural Analysis of Butyl-Fructooligosaccharides (B-FOS)

#### 2.1.1. Determination of Linkages in B-FOS by Fourier Transform Infrared (FT-IR) Spectroscopy Analysis

Fourier transform infrared (FT-IR) spectroscopy is generally utilized to collect an IR spectrum from a specimen and analyze the absorbed information by displaying it in spectral form. Thus, the chemical bonds in B-FOS and FOS were identified and compared via FT-IR spectroscopy analysis.

**Figure 1:**
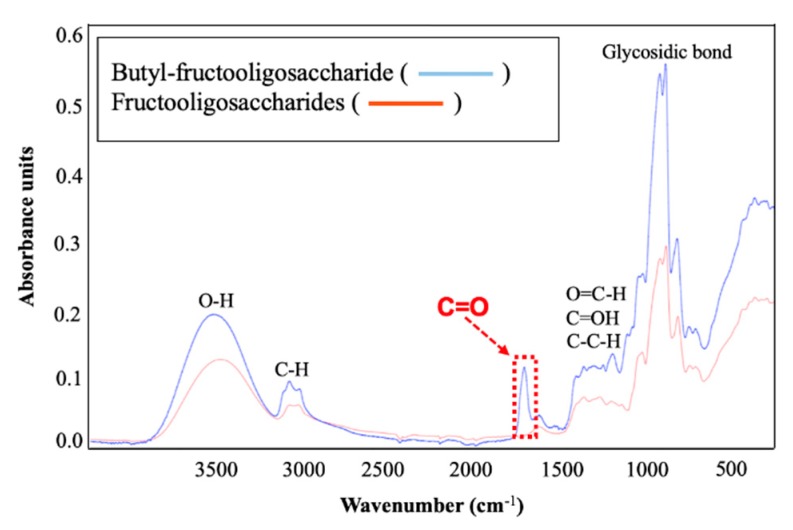
Absorbance spectra of butyl-fructooligosaccharides (B-FOS) and FOS by Fourier transform infrared (FT-IR) spectroscopy analysis. B-FOS (blue line); FOS (red line).

The results shown in Figure 1 display the absorbance spectra of the chemical bonds in B-FOS (blue line) and FOS (red line). Specifically, the broad bands in the 3000–3700, 2700–3000, and 1600–1630 cm^−1^ ranges observed in both B-FOS and FOS were assigned to hydroxyl groups (O–H) [20], hydrocarbon (C–H) and carbonyl groups (COO^−^) [21], respectively. In addition, bending vibration groups (OCH, COH, CCH) in the 1300–1500 cm^−1^ region [21] and stretching vibration of glycosidic bonds in the 800–1200 cm^−1^ region were also observed in both B-FOS and FOS curves [22]. Notably, the patterns of these two curves were highly similar, with the exception of the absorption at 1720 cm^−1^, which was only observed in the B-FOS band. Based on analysis of the spectra, the significantly high intensity of absorbance at 1720 cm^−1^ in the B-FOS band is due to the presence of an ester bond (C=O) [20]. Therefore, we hypothesize that butyrate can be linked to FOS via ester bonds to form the B-FOS structure.

#### 2.1.2. Mass Spectral Analysis by Matrix-Assisted Laser Desorption/Ionization Time-of-Flight (MALDI-TOF) Mass Spectrometry

**Figure 2:**
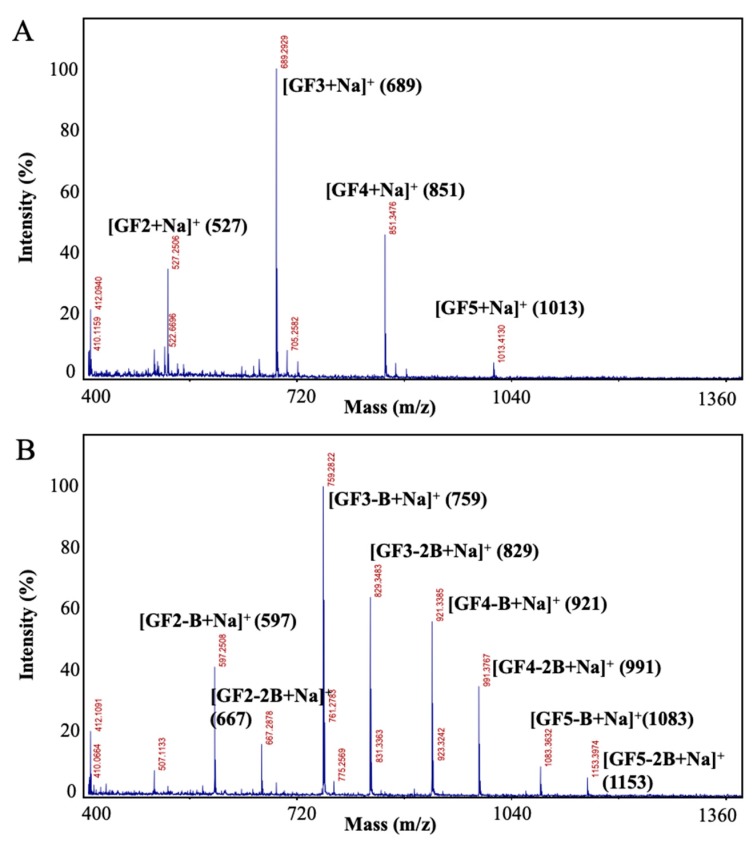
Molecular ion peaks of FOS and B-FOS by matrix-assisted laser desorption/ionization time-of-flight mass spectrometry (MALDI-TOF-MS). The mass spectra were obtained in the *m*/*z* range from 400 to 2000. (**A**) FOS; (**B**) B-FOS.

Matrix-assisted laser desorption/ionization time-of-flight mass spectrometry (MALDI-TOF MS), an important tool for observing the complexity and diversity of biological macromolecules [23], was applied to characterize and compare FOS and B-FOS profiles. The peaks corresponding to [M + Na]^+^ ions of FOS and B-FOS are shown in Figure 2. In this work, FOS has been used as an important substrate for the synthesis of B-FOS, with the components as follows: one (1-kestose, GF2: glucose–fructose–fructose), two (nystose, GF3: glucose–fructose–fructose–fructose), three (fructosyl nystose GF4: glucose–fructose–fructose–fructose) and/or four (fructosyl fructosylnystose, GF5: glucose–fructose–fructose–fructose–fructose) additional fructose units possibly linked to the sucrose by β (2–1) glycosidic bonds.

As shown in Figure 2A, the molecular weights of FOS were determined to be 504, 666, 828 and 990, based on the molecular ion peaks at *m*/*z* 527, 689, 851 and 1013 [M + Na]^+^, respectively. This suggests that FOS, as the substrate of B-FOS synthesis, is composed of GF2 GF3, GF4 and GF5. Also shown in Figure 2B, the molecular weights of B-FOS were determined to be *m*/*z* 597, 667, 759, 829, 921, 991, 1083 and 1153 [M + Na]^+^, respectively. From Figure 1 and Figure 2 it was determined that one or two butyrate(s) and FOS in B-FOS were combined by ester bonds. Furthermore, the intensities of molecular ion peaks indicated that GF3 was the major component of FOS, followed by GF4, GF2, and GF5. This composition was similar that of B-FOS. The ratio of B-FOS with one butyrate and B-FOS with two butyrates was approximately 5:3. In addition, none of the molecular ion peaks in FOS were detected (Figure 2B), which suggests that FOS was completely removed in the process of B-FOS purification.

#### 2.1.3. Nuclear Magnetic Resonance (NMR) Analysis

The ^1^H nuclear magnetic resonance (NMR) spectrum of B-FOS (Figure S2A) revealed the presence of one signal in the anomeric region at 5.44 ppm, which was the anomer proton (H-1) of the α-glucopyranose. Compared to FOS NMR data and previous publications [24,25], an oxygenated methylene (Fru-1) and an oxygenated methine (Fru-4) were detected at a lower magnetic field (*δ*_H_ 4.42 (Fru-1a), 4.29 (Fru-1b), and 5.25 (Fru-4), respectively) than the commonly detected chemical shift (*δ*_H_ 3.83 (Fru-1) and 4.13-4.03 (Fru-4)) due to an esterification shift, which confirmed the position of the ester linkages. Also in the ^13^C-NMR spectrum (Figure S2B), the presence of butyrate in B-FOS was confirmed.

**Figure 3:**
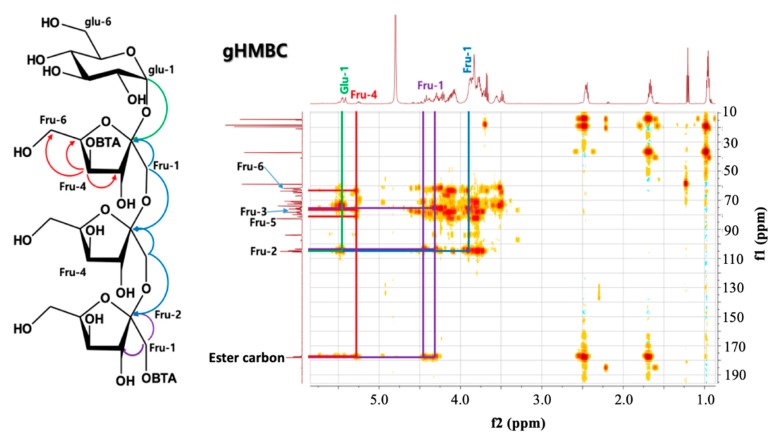
Two-dimensional nuclear magnetic resonance (2D-NMR) HMBC (heteronuclear multiple bond correlation) spectrum of B-FOS. The arrows indicate key correlations of B-FOS in the gHMBC spectrum. BTA denotes butyrate.

In the HMBC (heteronuclear multiple bond correlation) spectrum (Figure 3), H-1 (*δ*_H_ 5.25) and H-4 (*δ*_H_ 4.42) of a fructosyl residue in B-FOS are shown cross peak with the ester group. From this result, it was confirmed that butyrate(s) are attached to H-1 or H-4 of a fructofuranose in B-FOS. In addition, the integral value of the Fru-4 combined with butyrate was much smaller than that of the anomer proton of glucose (Figure S2), which further proved that B-FOS with two butyrates combined at Fru-4 and Fru-1 was a relative minor component. Taken together, the cross peaks of the H-1 proton signals (Figure 3) of glucopyranosyl and fructofuranosyl confirmed that the 1st butyrate is attached at H-1 of the terminal fructofuranose. However, which fructosyl residue was combined with the 2nd butyrate at H-4 in the second structure of B-FOS was unclear.

#### 2.1.4. Low-Resolution Fast-Atom Bombardment Mass Spectrometry (LR-FAB-MS) Analysis

**Figure 4:**
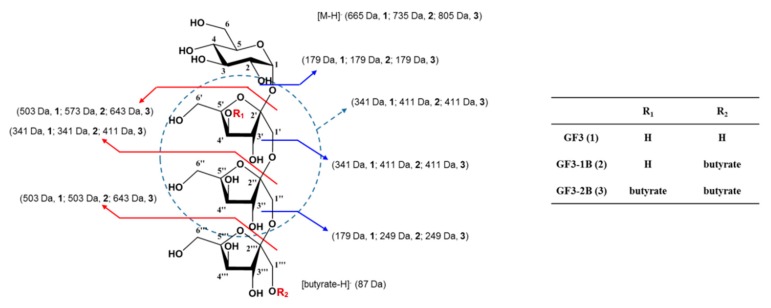
A proposed model of the major structure of the B-FOS. The site where the butyrate is attached to the fructosyl residue is marked with the R group.

The structure of B-FOS was further analyzed via identification of the second butyrate-linked fructosyl residues with negative fast-atom bombardment mass spectrometry (FAB-MS) analysis. The results of negative FAB-MS spectra of FOS and B-FOS are shown in Figure S3. The molecular ion peaks of FOS were observed at *m*/*z* 179, 341, 503, 665, while in B-FOS they were observed at *m*/*z* 179, 249, 341, 411, 573, 643, 735, 805. The ions in FOS were in accordance with the FOS structure. Due to the absence of ion peaks at *m*/*z* 319 and 481, it was confirmed that butyrate molecules were not bound to the last or second-to-last fructosyl residues. The mass spectra of fragments of the original structure indicated that butyrate at H-4 was combined with the first fructosyl residue in the structure of B-FOS with the 2nd butyrate. The ions observed at *m*/*z* 735 and 805 probably correspond to the deprotonated *O*-(1-butyratyl)-*β*-D-fructofuranosyl-(2→1)-*O*-*β*-D-fructofuranosyl-(2→1)-*O*-*β*-D-fructofuranosyl-*O*-*α*-D-glucopyranoside, and to the *O*-(1-butyratyl)-*β*-D-fructofuranosyl-(2→1)-*O*-*β*-D-fructofuranosyl-(2→1)-*O*-(4-butyratyl)-*β*-D-fructofuranosyl-*O-α*-D-glucopyranoside. Taken together, both major forms of B-FOS were confirmed, as shown in Figure 4.

The chemical structures of GF3, GF3-1B and GF3-2B were further identified by ^1^H-NMR (600 MHz, D_2_O, *δ*_H_) and ^13^C-NMR (150 MHz, D_2_O, *δ*_C_), molecular formulas were determined by Maldi-QTof-MS (matrix-assisted laser desorption ionization quadrupole time-of-flight-mass spectrometry), and molecular weight and fragments were determined by FAB-MS. The results were shown as: (i) *O*-*β*-D-fructofuranosyl-(2→1)-*O*-*β*-D-fructofuranosyl-(2→1)-*O*-*β*-D-fructofuranosyl-*O*-*α*-D-glucopyranoside (1; GF3): negative FAB-MS *m*/*z* 665 [M − H]^−^, 503 [M-fructofuranose-H or M-glucopyranose-H]^−^, 341 [fructofuranosyl glucopyranoside-H or fructofuranosyl fructofuranoside-H]^−^, 179 [glucopyranose-H or fructofuranose-H]^−^; Maldi-QTof-MS *m*/*z* 689.2929 [M + Na]^+^ (calcd. for C_24_H_42_NaO_21_, 689.2116). (ii) *O*-(1-butyratyl)-*β*-D-fructofuranosyl-(2→1)-*O*-*β*-D-fructofuranosyl-(2→1)-*O*-*β*-D-fructofuranosyl-*O*-*α*-D-glucopyranoside (2; GF3-1B): negative FAB-MS *m*/*z* 735 [M − H]^−^, 573 [M-glucopyranose-H]^−^, 411 [M-fructofuranosyl glucopyranoside-H]^−^, 341 [fructofuranosyl glucopyranoside-H or fructofuranosyl fructofuranoside-H]^−^, 249 [M-fructofuranosyl fructofuranosyl glucopyranoside-H]^−^, 179 [glucopyranose-H or fructofuranose-H]^−^, 87 [butyrate-H]^−^; Maldi-QTof-MS *m*/*z* 759.2535 [M + Na]^+^ (calcd. for C_28_H_48_NaO_22_, 759.2822). (iii) *O*-(1-butyratyl)-*β*-D-fructofuranosyl-(2→1)-*O*-*β*-D-fructofuranosyl-(2→1)-*O-*(4-butyratyl)-*β*-D-fructofuranosyl-*O*-*α*-D-glucopyranoside (3; GF3-2B): negative FAB-MS *m*/*z* 805 [M − H]^−^, 643 [M-glucopyranose-H]^−^, 573 [M-fructofuranose-butyrate-H]^−^, 411 [M-fructofuranosyl glucopyranoside-butyrate-H or M-fructofuranosyl fructofuranoside-butyrate-H]^−^, 341 [fructofuranosyl glucopyranoside-H or fructofuranosyl fructofuranoside-H]^−^, 249 [M-fructofuranosyl fructofuranosyl glucopyranoside-butyrate-H]^−^, 179 [glucopyranose-H or fructofuranose-H]^−^, 87 [butyrate-H]^−^; Maldi-QTof-MS *m*/*z* 829.3483 [M + Na]^+^ (calcd. for C_32_H_54_NaO_23_, 829.2954). ^1^H-NMR (600 MHz, D_2_O, *δ*_H_) and ^13^C-NMR (150 MHz, D_2_O, *δ*_C_) data of experimental compounds (i.e., GF3, GF3-1B, GF3-2B) are featured in Table 1 and Table 2.

### 2.2. Fecal Batch Culture Fermentation

#### 2.2.1. Fecal Butyrate Level Changes by B-FOS Treatment

To verify potential B-FOS biofunctionality as a prebiotic, SCFA levels in fecal samples were examined in vitro. The changes in SCFA concentration during fermentation are presented in Table 3. Butyrate, the SCFA focused upon in this study, was not detected before fermentation. After 24 h of anaerobic fermentation, the levels of butyrate in the feces increased in all groups. The amount of butyrate in the B-FOS treated group increased most significantly after fermentation (*p* < 0.05, *n* = 3). In the FOS treatment group, acetate increased most significantly after fermentation compared to other groups (*p* < 0.05, *n* = 3).

#### 2.2.2. Bacterial Diversity

**Figure 5:**
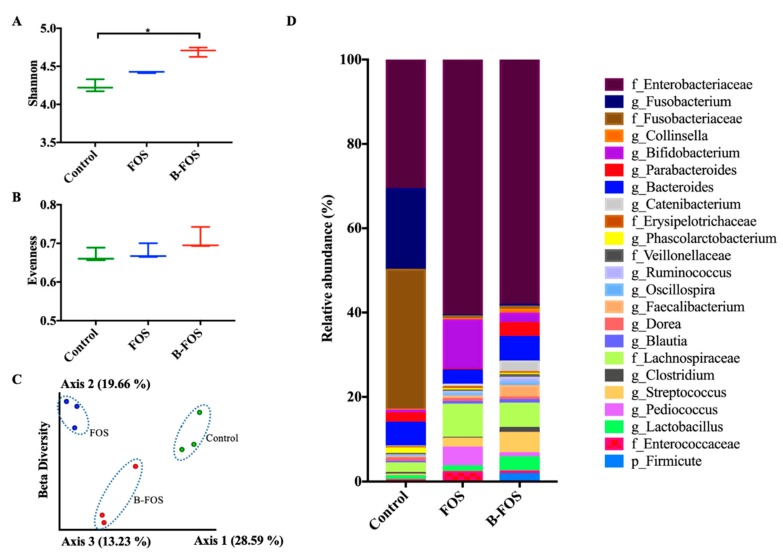
Comparison of diversity indices amongst groups after 24 h fermentation. Alpha diversity of microbial communities are shown as richness (**A**) and evenness (**B**). (**C**) Beta diversity analyzed by Jaccard. (**D**) Average relative abundance at the genus level. Taxa with relative abundance below 0.5% were excluded prior to analysis. Significance was accepted at * *p* < 0.05 (*n* = 3).

Alpha diversities were evaluated via richness (shannon) and evenness analyses. Richness is a measure of the number of species and evenness is the degree of homogeneity of species identified in the sample [26]. As shown in Figure 5A, the mean alpha diversity of richness in the B-FOS group was significantly larger than the control (*p* < 0.05). Beta diversity measurements were performed to evaluate the degree of change in natural flora composition among the three groups (i.e., control, B-FOS treated and FOS treated groups) by Jaccard dissimilarity distance analysis (Figure 5C). Each of the three bacterial groups showed distinct clustering in microbial communities. The average relative abundances at the genus level of the three groups displayed in Figure 5D also indicate the different microbial compositions amongst the groups after fermentation.

#### 2.2.3. Microbiota Composition

**Figure 6:**
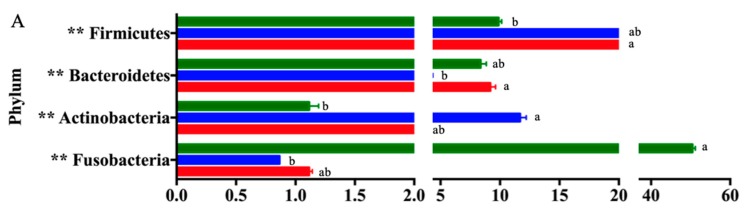

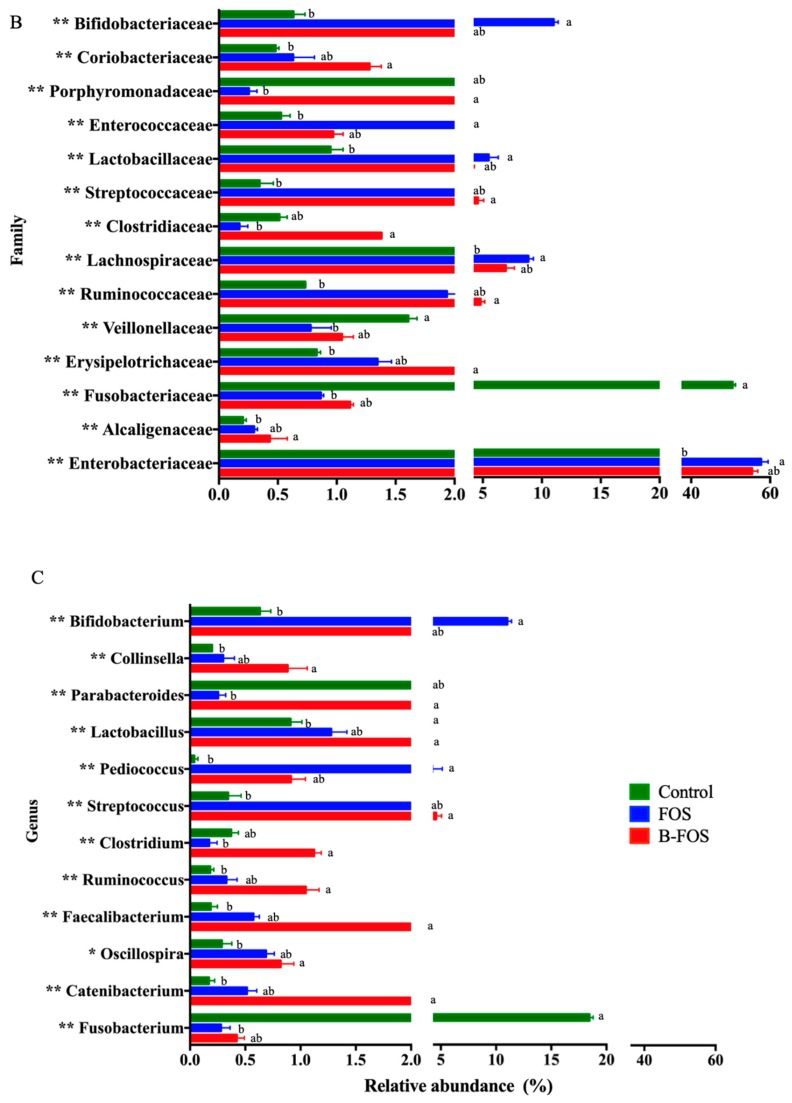
Taxonomic differences in fecal microbiota. Comparison of relative abundance at the bacterial phylum (**A**), family (**B**) and genus levels (**C**). Error bars represent means ± SD of triplicate cultures. Treatments with different letters are significantly different at *p* < 0.05. Relative abundance >0.5%. * *p* < 0.05, ** *p* < 0.01.

To examine the effect of B-FOS on compositional changes in the gut microbiota, we identified the significantly different phylotypes among the groups and the key phylotypes responsible for the differences among the control group, FOS group and B-FOS group after fermentation (Figure 6).

Statistically significant differences among the three groups were observed with respect to the four dominant phyla: Firmicutes, Bacteroidetes, Actinobacteria, Fusobacteria. In addition, 14 statistically significant differences amongst the groups were identified at the family level (Figure 6B). The relative abundances of Coriobacteriaceae, Porphyromonadaceae, Streptococcaceae, Clostridiaceae, Ruminococcaceae, Erysipelotrichaseae and Alcaligenaceae were significantly abundant in the B-FOS treatment, compared to the other groups, after fermentation. Meanwhile, the proportions of Bifidobacteriaceae, Enterococcaceae, Lactobacillaceae, Lachnospiraceae and Enterobacteriaceae in the FOS treatment were significantly larger than in the other groups. Also, a significantly lower level of Fusobacteriaceae was observed in the B-FOS treatment than in the control, after fermentation. At the genus level, 14 genera were found to be significantly different among the groups (Figure 6C). *Collinsella*, *Parabacteroides*, *Lactobacillus*, *Streptococcus*, *Clostridium*, *Ruminococcus*, *Faecalibacterium*, *Oscillospira*, and *Catenibacterium* were relatively more abundant in the B-FOS treatment than in the other groups. Meanwhile, *Bifidobacterium* and *Pediococcus* were most abundant in the FOS treatment. It is notable that the proportion of *Fusobacterium* significantly increased in the control group compared with the other two groups.

#### 2.2.4. Butyrate-Producing Bacteria

Multiple groups have reported that *Faecalibacterium*, *Eubacterium*, *Ruminococcus*, *Clostridium*, *Roseburia*, *Butyrivibrio*, *Anaerostipes*, *Coprococcus*, *Oscillospira*, *Collinsella* etc. are major butyrate-producing microorganisms [27,28,29,30,31]. In this study, *Ruminococcus*, *Clostridium*, *Oscillospira*, *Collinsella* and *Faecalibacterium* were selected, and bacteria with relative abundances below 0.5% were excluded.

The relative abundances of butyrate-producing bacteria (*Faecalibacterium*, *Ruminococcus*, *Clostridium*, *Oscillospira*, *Collinsella*) in the B-FOS group were maintained at the level before the fermentation. The relative abundances of butyrate-producing bacteria in the control or FOS group sharply decreased after 24 h fermentation (Table 4, *p* < 0.05).

#### 2.2.5. Microbial Function Analysis to Investigate the Butyrate Production Pathway

**Figure 7:**
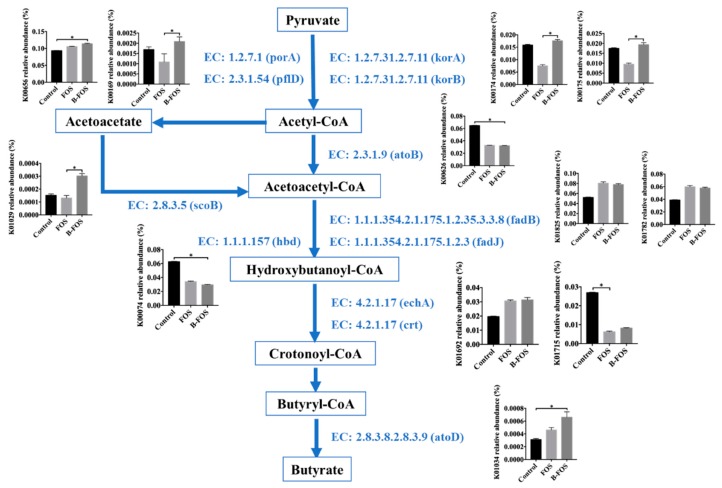
The butyrate production pathway in commensal butyrate-producing bacteria. Related enzymes involved 2-oxoacid ferredoxin oxidoreductase subunit alpha (korA), 2-oxoacid ferredoxin oxidoreductase subunit beta (korB), pyruvate ferredoxin oxidoreductase alpha subunit (porA), formate C-acetyltransferase (pflD), acetyl-CoA C-acetyltransferase (atoB), 3-hydroxyacyl-CoA dehydrogenase (fadB), 3-hydroxyacyl-CoA dehydrogenase (fadJ), 3-hydroxybutyryl-CoA dehydrogenase (hbd), enoyl-CoA hydratase (echA), enoyl-CoA hydratase (crt), acetate CoA/acetoacetate CoA-transferase alpha subunit (atoD), 3-oxoacid CoA-transferase subunit B (scoB). Arrows indicate the related genes that are involved in the corresponding pathway. Error bars represent means ± SD of triplicate cultures. Significance was accepted at * *p* < 0.05 (*n* = 3).

To investigate the role of the commensal butyrate-producing bacteria in the B-FOS group, Kyoto Encyclopedia of Genes and Genomes (KEGG) analysis by phylogenetic investigation of the community by reconstruction of unobserved states (PICRUSt) was carried out (Figure 7). After comparing all the KEGG genes presented in butanoate metabolism amongst the groups, the KEGG genes that were significantly different within the groups are displayed in Figures S4–S6.

According to the butyrate pathway shown in Figure 7, B-FOS significantly increased the relative abundances of porA, pflD, korA and korB, which converted pyruvate into acetyl-CoA. The relative abundances of scoB and atoD were also significantly abundant in the B-FOS group, which were responsible for the conversion from acetyl-CoA to acetoacetyl-CoA via the intermediate acetoacetate and the conversion from butyryl-CoA to butyrate, respectively. Meanwhile, the relative abundances of porA, korA, korB and scoB were positively correlated with butyrate production, as shown in Figure S7 (*p* < 0.05).

#### 2.2.6. Ammonia Analysis

**Figure 8:**
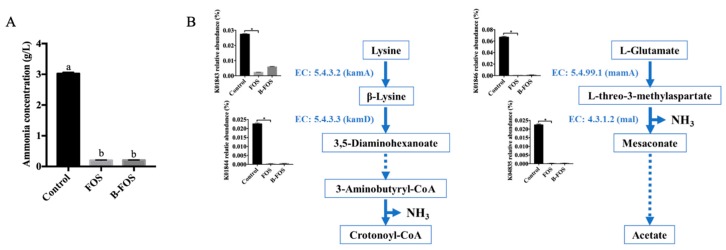
(**A**) Ammonia concentrations in fecal broth after 24 h culture. (**B**) Ammonia production pathways in *Fusobacterium*. Lysine 2,3-aminomutase (kamA), beta-lysine 5,6-aminomutase (kamD), methylaspartate mutase (mamA), methylaspartate ammonia lyase (mal). Arrows indicate the related genes that are involved in the corresponding pathway. Error bars represent means ± SD of triplicate cultures. Treatments with different letters are significantly different at *p* < 0.05. Significance was accepted at * *p* < 0.05 (*n* = 3).

Ammonia is one of the fermentation by-products that adversely affects host health and has toxic effects on the large intestine [32,33]. Figure 8A shows the level of ammonia produced during fermentation with or without the addition of B-FOS or FOS. The concentration of ammonia was undetectable at 0 h. However, the level of ammonia in fecal samples were significantly increased in the control group after 24 h incubation (*p* < 0.05, *n* = 3), while ammonia was barely detected in the B-FOS and FOS treatment groups.

*Fusobacterium* consumed amino acids (lysine and glutamate) and released ammonia as a by-product of butyrate production. KEGG analysis associated with ammonia release indicated that the relative abundances of *kamA, kamD, mamA* and *mal* were significantly increased in the control (Figure 8), which indicates the consumption of lysine and glutamate was stimulated. Moreover, the relative abundances of these genes were positively correlated to ammonia concentration and the relative abundance of *Fusobacterium* (Figure S8). Consequently, the significantly high ammonia level observed in the control was closely linked with the increase of *Fusobacterium* abundance.

## 3. Discussion

B-FOSs are newly synthesized compounds combining FOSs with butyrate. FT-IR and MALDI-TOF-MS analysis of B-FOS showed they were combined with one or two butyrate molecule(s) by ester bonds. As FOSs are composed of GF2, GF3, GF4 and GF5, B-FOS correspondingly consists of GF2-GF5 with butyrate(s). GF3-1B and GF3-2B, as the major components, were selected to further deduce B-FOS structures via 1D and 2D NMR and FAB-MS. The major B-FOS structures are GF3-1B [*O*-(1-buty-*β*-D-fru-(2→1)-*O*-*β*-D-fru-(2→1)-*O*-*β*-D-fru-*O*-*α*-D-glu] and GF3-2B [*O*-(1-buty)-*β*-D-fru-(2→1)-*O*-*β*-D-fru-(2→1)-*O-*(4-buty)-*β*-D-fru-*O*-*α*-D-glu]. The ratio of these two compounds was approximately 5:3, based on MALDI-TOF-MS and NMR analysis. The structural characteristics of B-FOS appear to be advantageous in terms of gut microbiota remodeling and butyrate production.

To evaluate the prebiotic properties of B-FOS, its effects on gut microbiota were investigated using mixed fecal batch cultures, which can offer valuable insights and provide for rapid evaluation of prebiotics before conducting in vivo experiments [34]. This study showed that B-FOS increased butyrate concentration, inhibited ammonia production, and modulated intestinal bacteria compositions via different mechanisms by FOS.

Microbial community changes were explored by 16S rRNA community analysis. The richness of gut microbe diversity was significantly larger in the B-FOS group than the control and the beta diversity showed different microbial compositions amongst the groups, which suggests different effects of B-FOS and FOS on gut microbiota remodeling. As the most typical probiotic organisms, the growth of *Bifidobacterium* and *Lactobacillus* were investigated amongst the groups to evaluate the potential prebiotic effects of B-FOS. The relative abundance of *Bifidobacterium* was significantly more abundant in the FOS group (11.02%), while the levels of *Bifidobacterium* in the B-FOS group and control were only 2.14% and 0.063%, respectively. Based on the taxa profile, *Lactobacillus* was one of the significantly abundant phylotypes in the B-FOS group (3.17%), while the proportions in the control and FOS group were 0.91% and 1.28%, respectively. In addition, it is recognized today that prebiotic effects related to selective stimulation extend beyond *Bifidobacterium* and *Lactobacillus* [35]. The high diversity in the microbial composition with B-FOS treatment supports its potential for use as a novel prebiotic.

B-FOS also significantly increased the proportions of *Parabacteroides* (3.13%), *Oscillospira* (0.82%), *Catenibacterium* (2.41%), *Streptococcus* (4.59%), *Faecalibacterium* (2.59%), *Clostridium* (1.12%), *Ruminococcus* (1.06%) and *Collinsella* (0.88%) at the genus level. Consistent with our results, a significant reduction of *Parabacteroides* after FOS intervention was observed in a mouse model study conducted by Gu et al. [36]. *Parabacteroides* has been repeatedly linked with metabolic health [37,38,39,40]. The low level of *Parabacteroides* has been observed in patients with obesity, non-alcoholic fatty liver and multiple sclerosis. Interestingly, the abundances of *Parabacteroides, Catenibacterium* and *Oscillospira* have been reported to be significantly negatively correlated with body mass index (BMI) [40,41,42]. *Catenibacterium* is also negatively associated with lifetime cardiovascular disease (CVD) risk profiles [43]. In addition, *Collinsella* has been reported to modify host bile acids and plasma cholesterol levels [31]. Another significant increase observed in the B-FOS group was in *Streptococcus*. Even though some important pathogens belong to the genus *Streptococcus*, many species of *Streptococcus* in the intestine are commensals [44]. As a result, the genera associated with metabolism regulation (*Parabacteroides*, *Catenibacterium*, *Oscillospira* and *Collinsella*) were significantly more abundant in the B-FOS group.

The primary cause of the butyrate surge observed in the B-FOS treatment could be the release of butyrate from B-FOS by cleavage and microbial production of butyrate during fermentation. To investigate the sources of butyrate generation, the relative abundances of butyrate-producing commensal bacteria were examined. The relative proportions of the selected butyrate-producing commensal bacteria (*Faecalibacterium*, *Clostridium* genera, *Ruminococcus, Oscillospira* and *Collinsella*) were maintained in the B-FOS group and were reduced in the control and FOS groups after fermentation. This suggests the capability of B-FOS to protect the growth of butyrate-producing commensal bacteria.

The results related to butyrate-producing bacteria and butyrate production in the FOS group were consistent with a random, double-blind cross-over study performed by Liu et al., (2017) [45]. This evaluation of prebiotic intervention in a healthy, young population indicated that FOS intervention significantly increased *Bifidobacterium* and significantly decreased butyrate-producing bacteria (including *Ruminococcus* and *Oscillospira*) after prebiotic intervention. Several in vitro fecal fermentation studies have also indicated that the addition of FOS significantly decreases the abundance of *Faecalibacterium* [46,47]. *Faecalibacterium*, abundant in the B-FOS group, can serve as an indicator or biomarker of intestinal health in adults [48]. As a next-generation probiotic, it has been shown to be responsible for anti-inflammatory activity in the gut [49]. *Faecalibacterium* is exhausted in inflammatory bowel disease patients, especially in cases of Crohn′s disease, and its replacement has been considered as a potential therapeutic method in many studies [50]. Another novel butyrate-producing bacterium, *Collinsella*, was also noted for its potential for treatment of inflammatory bowel disease [51].

Butanoate metabolism was explored via KEGG analysis by PICRUSt. Pyruvate was utilized as the initial substrate to produce butyrate by commensal butyrate-producing bacteria [52]. B-FOS probably stimulated the butyrate production by significantly increasing the conversion from pyruvate to acetyl-CoA via porA, korA and korB, which were positively correlated with butyrate generation.

Notably, the genus *Fusobacterium* (belonging to the family Fusobacteriaceae) was drastically increased (from 1.28% to 18.45%) in the control after the 24-h fermentation. The initial relative proportions of *Fusobacterium* were only 0.28% and 0.42% in the FOS and B-FOS group, respectively. *Fusobacterium* are considered to be ammonia-producing butyrogenesis gut pathogens [52,53]. Lysine and glutamate are likely to be utilized by *Fusobacterium*. The high relative abundances of *kamA* and *kamD* in the control suggests a greater conversion from lysine to 3,5-diaminohexanoate, which might lead to larger ammonia production downstream in the pathway. Similarly, the control promoted the alteration of glutamate into mesaconate via *mamA* and *mal*, with the release of ammonia in the process. This, coupled with the positive correlations between these genes and ammonia/*Fusobacterium*, indicate that the significantly higher concentration of ammonia and relatively high butyrate concentration in the control was caused by the high relative abundance of *Fusobacterium*. B-FOS and FOS may potentially inhibit the increase of ammonia in vitro by inhibiting the growth of *Fusobacterium*. This result is consistent with previous FOS-related studies which indicate that supplementation of the diet with FOS reduces levels of *Fusobacterium* [54], and administration of FOS decreases ammonia concentrations, based on an in vitro canine fecal fermentation study [55].

Usually, butyrate molecules are produced from carbohydrates by commensal butyrate-producing bacteria via the pyruvate pathway. Condensation of two acetyl-CoA molecules produces an acetoacetyl-CoA molecule, followed by the butyryl-CoA reduction process [56]. Unlike these commensal bacteria, *Fusobacterium* utilized lysine and glutamate pathways for butyrate production, accompanied by ammonia release [57]. Although differences in the butyrogenic effects on gut health between commensal and pathogen bacteria have not yet been reported, this butyrate-producing pathogen can compete for essential amino acids during butyrate generation and the release of ammonia may cause gut integrity damage and further increase the probability of pathogen invasion [52]. Recent studies have reported a positive correlation between *Fusobacterium* abundance and IBD in the host [58], and a strong association between *Fusobacterium* and colorectal cancer (CRC) [59]. Moreover, *Fusobacteirum* was the only pathogen with butyrate-producing abilities that was observed amongst all highly abundant pathogens found in IBD and CRC cohorts [52].

The prebiotic effects of B-FOS on gut microbiota were evaluated in an in vitro model in this study. Experimental control and repeatability is better in the in vitro experiments vs. in the in vivo conditions, and costs are lower, in addition, but in vitro fecal batch fermentation also has limitations. Promotion of selected bacterial species and accumulation of their metabolite products (i.e., SCFA) results in pH reduction during fermentation, which impacts the microbial composition. The lack of pH control is considered as a general phenomenon for in vitro batch fermentation experiments [35]. Nonetheless, previous studies have reported that the pH is lowest in the proximal colon (~5.6) and increases towards the distal colon (~6.3) [1,60], which indicates that the pH changes in this study are within a reasonable range. Although the host colonic environment cannot be completely reproduced in the in vitro studies, in vitro models are practical tools for the investigation of the alteration of microbiota in response to exposure to different compounds [61]. Another limitation in this study was that we could not distinguish the butyrate produced via butyrate-producing bacteria vs. that resulting from B-FOS breakdown.

Taken together, the microbiota changes, butyrate production and ammonia reduction associated with B-FOS suggest its prebiotic potential. The differences in the 16S metagenomic results of B-FOS and FOS treatments indicate that B-FOS alters the intestinal microbiota differently than FOS. Many representative bacteria observed in the B-FOS treatment have been linked to metabolism regulation, anti-inflammation or butyrate generation, which may bring more benefits than FOS. Considering the limitations of in vitro fermentation modeling, claims of B-FOS prebiotic effects should be evaluated by well-designed animal experiments and clinical studies. It is expected that B-FOS fermentation in vivo may display more effective results due to metabolism. More importantly, as a more palatable butyrate-releasing compound, oral B-FOS administration may be expected to result in greater patient compliance as well as more effective butyrate delivery and, therefore, be preferred over traditional butyrate administration. Therefore, additional B-FOS studies will be conducted in the healthy mouse gut, mouse metabolic disfunction models, and mouse colitis models, and further investigated via double-blind controlled clinical trials to test its effectiveness via oral administration.

## 4. Materials and Methods

### 4.1. Preparation of B-FOS

B-FOS was produced by Bifido Inc. (Gangwon-do, Korea). It was synthesized by adding butyrate (Sigma-Aldrich, St. Louis, MO, USA) to an aqueous solution of >90% pure fructooligosaccharides (FOS, Samyang Corp., Seoul, Korea). FOS and butyric anhydride 98% (Sigma-Aldrich, St. Louis, MO, USA) were used in the B-FOS synthesis process. FOS (50% *w*/*v*) was mixed with butyrate in a ratio of 10: 1, at a temperature of 50 °C for 3 h. The product was neutralized with a NaOH solution to complete the reaction. The purification of B-FOS was performed by filtration through synthetic absorbent Diaion HP20 packed in a 50 × 5 cm Glass Econo-Column chromatography column (Bio-Rad, Hercules, CA, USA). The 40%–70% (*v*/*v*) ethanol eluent was collected and the purity of B-FOS was tested by thin-layer chromatography (TLC) with a solvent of 1-propanol/water/ethyl acetate (7:2:1, *v*/*v*) (See Figure S1). Purified B-FOS was concentrated via a speed vacuum concentrator (ScanSpeed 40, Labogene, Lynge, Denmark) and freeze-dried (Ilshin Biobase, Yangju, Korea). The final B-FOS product was a soluble carbohydrate powder.

### 4.2. Structural Analysis of B-FOS

The powder-formed, purified B-FOS and FOS were examined by FT-IR spectroscopy (Bruker Optics, Ettlingen, Germany) using a single-reflection attenuated total reflection (ATR) attachment equipped with a Di Crystal. Depth penetration during the analysis was estimated to be about 2 microns. The spectrum was collected using a TENSOR27/Bruker with 128 scans at 4 cm^−1^ resolution. Mass spectra of FOS and B-FOS were analyzed by MALDI-TOF-MS and LR-FAB-MS. MALDI-TOF-MS was conducted using a Voyager-DETM STR Biospectrometry Workstation (Applied Biosystems, Foster City, CA, USA). The matrix substance was 2,5-dihydroxybenzoic acid. The mass spectra of FOS and B-FOS were measured in the *m*/*z* range from 400 to 2000. LR-FAB-MS was conducted using a JMS-700 (JEOL, Tokyo, Japan) in the negative ion mode, with glycerol as the matrix and xenon gas. Mass ranges were from *m*/*z* 10 to 2000. 1D (^1^H and ^13^C) and 2D (gHMBC) NMR spectra, were measured using a 600 MHz NMR instrument (Bruker Daltonik, Bremen, Germany) and analyzed by BBIOREFCODE pH7. Samples were prepared in D2O at a concentration of 20 mg/mL. Chemical shifts were expressed in parts per million (ppm) relative to 2,2,3,3-tetradeuterio-3-(trimethylsilyl)-propanoic acid sodium salt, which was used as an internal chemical shift reference at 0 ppm [25]. MestReNova software (version 5.3.1-4696, Santiago de Compostela, Spain) was utilized to enhance resolution of experimental results.

### 4.3. Fecal Batch Culture Fermentation

The method of fecal batch culture fermentation used has been described previously [62,63,64]. Nine 10 mL autoclaved serum bottles were filled with 5 mL of brain heart infusion broth without dextrose (MB cell, Seoul, Korea). The pH was adjusted to 7.0 with aqueous 5 N NaOH and autoclaved at 121 °C for 15 min. B-FOS or FOS was added as the sole carbon source at a concentration of 1% (*w*/*v*). FOS treatment was used as a positive fermenting control. A 10% (*w*/*v*) fecal slurry was prepared using fresh feces (details of collection provided below) mixed with 0.1M phosphate-buffered saline (pH 7.0) in a stomacher for 2 min. Each bottle was inoculated with 5 mL of fecal slurry to give a final concentration of 5% (*w*/*v*). Each fermentation (1 mL) was kept in a 1.5 mL Eppendorf tube for SCFA analysis and ammonia measurement at 0 h. The fermentations were then capped with silicon plugs, sealed with aluminum caps, and incubated at 37 °C for 24 h [65]. The experiments were conducted in an anaerobic chamber (Coy Lab, Grass Lake, MI, USA) to minimize the oxygen content at the initial time point. Experiments were run in triplicate, with fecal samples obtained from five healthy 20- to 30-year-old male and female volunteers who had no medical history related to gastrointestinal disorders and had not taken any antibiotics for at least 3 months prior to providing the samples. Fresh feces were collected and immediately stored at −80 °C until used. Written informed consent was received from all volunteers before collection. The study was conducted in accordance with the Declaration of Helsinki, and the protocol was approved by the Ethics Committee of Seoul National University.

#### 4.3.1. Short-Chain Fatty Acids (SCFA) Analysis Using High-Performance Liquid Chromatography (HPLC)

One mL of each fermentation was transferred to a 1.5 mL Eppendorf tube and centrifuged at 18,000× g for 15 min. Supernatant fractions were filtered using 0.2 μm syringe filters and injected (20 μL) into a high-performance liquid chromatography (HPLC) system [62]. The YL9100 HPLC was equipped with an YL9170 RI detector and Younglin Autochro-3000 data system software (Younglin, Anayang, Korea). SCFAs were separated on an Aminex HPX-87H Ion Exclusion column (300 × 7.8 mm, 9 μm, Bio-Rad, USA) and run isocratically with 5 mM sulfuric acid (Samchun, Yeosu, Korea) at a flow rate of 0.6 mL per min and a temperature of 35 °C. The concentration of butyrate was measured and recorded.

#### 4.3.2. Bacterial Enumeration Using 16s Metagenome

DNA from the fecal fermentation samples was extracted using a QIAamp DNA Stool Mini Kit (Qiagen, UK) according to the manufacturer′s instructions. After extraction, DNA samples were stored at −20 °C prior to Next Generation Sequencing (NGS). Sample DNA concentrations were measured using a Qubit 3.0 Fluorometer (Thermo Fisher Scientific, Waltham, MA, USA) and diluted to 5 ng/μL. Genomic DNA was amplified using interest-specific primers with overhang adapters targeting the variable V3 and V4 region of the 16S rRNA. V3 and V4 amplicons of free primers and primer dimer species were purified by AMPure XP beads (Beckman Coulter, USA) and amplified with dual-index primers via PCR (forward primer, 5’ TCGTCGGCAGCGTCAGATGTGTATAAGAGACAGCCTACGGGNGGCWGCAG; reverse primer, 5′ GTCTCGTGGGCTCGGAGATGTGTATAAGAGACAGGACTACHVGGGTATCTAATCC). The amplicons were attached to specific barcode sequences to compile the pooled library. Five μL aliquots of diluted DNA (4 nM) were mixed for pooling libraries. A pooled library was denatured with NaOH and diluted to 9 pM with a hybridization buffer (HT1, Illumina, San Diego, CA, USA). A PhiX control library (Illumina, San Diego, CA, USA) was denatured with NaOH and diluted to 9 pM with HT1. The pooled library was mixed with the Phix control (30%, *v*/*v*) and loaded on a MiSeq v2 (500 cycle) Reagent cartridge (Illumina, San Diego, CA, USA) for sequencing Miseq instrument loading. Paired-end FASTQ files from NGS (next-generation sequencing) were imported into Quantitative Insights Into Microbial Ecology 2 (QIIME2) (ver. 2019.7, https://qiime2.org). Sequences were filtered, trimmed, de-noised, and merged using the DADA2 plugin. Chimeric sequences were identified and removed via the consensus method in the DADA2 plugin. Taxonomy was assigned to all ribosomal sequence variants in QIIME2 using a feature classifier trained with Greengenes 13_8 99% OTUs full-length sequences. Alpha and beta diversity analysis were performed using the q2-diversity plugin within QIIME2. PICRUSt was conducted to identify KEGG metabolic pathways potentially affected by different microbial communities.

#### 4.3.3. Ammonia Analysis

Ammonia concentrations of the fermentations were analyzed using the method described by Chaney et al. [66]. This method involved reactions with 10 g/L phenol (Junsei Chemical, Tokyo, Japan), 0.05 g/L sodium nitroprusside (Sigma-Aldrich, St. Louis, MO, USA), 5.0 g/L sodium hydroxide (Sigma-Aldrich, St. Louis, MO, USA), and 0.42 g/L sodium hypochlorite (Sumchun, Yeosu, Korea). Blue color produced after 30 min at room temperature was measured at 625 nm.

### 4.4. Statistical Analysis

Taxon-relative abundances were generated by the number of operational taxonomic units (OUT) divided by the total sum. Differential abundance analysis was performed by the Kruskal–Wallis test in a non-parametric one-way analysis of variance (ANOVA) test with Dunn′s multiple comparison test. Correlation analysis was conducted by the Spearman correlation test. The SCFA and ammonia analyses were carried out by one-way ANOVA with Tukey’s multiple comparisons test. All statistical analyses were performed using Graph-Pad Prism 8 (*p* < 0.05).

## Figures and Tables

**Table 1:** ^1^H-NMR data of fructooligosaccharide (GF3) and butyl-fructooligosaccharides (GF3-1B and GF3-2B) (*δ*_H_ in ppm).

No.	GF3	GF3-1B	GF3-2B
1	5.44, 1H, d, *J* = 4.2 Hz	5.43, 1H, d, *J* = 4.2 Hz	5.43, 1H, d, *J* = 4.2 Hz
2	3.56, 1H, dd, *J* = 8.4, 4.2 Hz	3.56, 1H, dd, *J* = 8.4, 4.2 Hz	3.56, 1H, dd, *J* = 8.4, 4.2 Hz
3	* 3.57–3.55, 1H	* 3.57–3.55, 1H	* 3.57–3.55, 1H
4	* 3.57–3.55, 1H	* 3.57–3.55, 1H	* 3.57–3.55, 1H
5	3.48, 1H, dd, *J* = 8.4, 8.4 Hz	3.48, 1H, dd, *J* = 8.4, 8.4 Hz	3.48, 1H, dd, *J* = 8.4, 8.4 Hz
6	* 3.83–3.63, 2H	* 3.83–3.63, 2H	* 3.83–3.63, 2H
1′	* 3.86, 2H	* 3.86, 2H	* 3.86, 2H
2′	-	-	-
3′	* 4.32–4.19, 1H	* 4.32–4.19, 1H	* 4.32–4.19, 1H
4′	* 4.13–4.03, 1H	* 4.13–4.03, 1H	* 5.25, 1H
5′	* 3.88, 1H	* 3.88, 1H	* 3.88, 1H
6′	* 3.83–3.63, 2H	* 3.83–3.63, 2H	* 3.83–3.63, 2H
1″	* 3.83, 2H	* 3.83, 2H	* 3.83, 2H
2″	-	-	-
3″	* 4.32–4.19, 1H	* 4.32–4.19, 1H	* 4.32–4.19, 1H
4″	* 4.13–4.03, 1H	* 4.13–4.03, 1H	* 4.13–4.03, 1H
5″	* 3.88, 1H	* 3.88, 1H	* 3.88, 1H
6″	* 3.83–3.63, 2H	* 3.83–3.63, 2H	* 3.83–3.63, 2H
1‴	* 3.83, 2H	* 4.42, 1H; 4.29, 1H	* 4.42, 1H; 4.29, 1H
2‴	-	-	-
3‴	* 4.32–4.19, 1H	* 4.32–4.19, 1H	* 4.32–4.19, 1H
4‴	* 4.13–4.03, 1H	* 4.13–4.03, 1H	* 4.13–4.03, 1H
5‴	* 3.88, 1H	* 3.88, 1H	* 3.88, 1H
6‴	* 3.83–3.63, 2H	* 3.83–3.63, 2H	* 3.83–3.63, 2H
buty-1		-	-
buty-2		2.46, 2H, m	2.46, 2H, m
buty-3		1.67, 2H, m	1.67, 2H, m
buty-4		0.96, 3H, t, *J* = 7.2 Hz	0.96, 3H, t, *J* = 7.2 Hz
buty-1′			-
buty-2′			2.46, 2H, m
buty-3′			1.67, 2H, m
buty-4′			0.96, 3H, t, *J* = 7.2 Hz

* overlapped.

**Table 2:** ^13^C-NMR data of fructooligosaccharide (GF3) and butyl-fructooligosaccharides (GF3-1B and GF3-2B) (*δ*_H_ in ppm).

No.	GF3	GF3-1B	GF3-2B
1	92.4	92.8	92.8
2	69.1	69.5	69.5
3	72.5	72.7	72.7
4	72.3	72.7	72.7
5	71.0	71.5	71.5
6	60.0	60.3	60.3
1′	62.1	62.4	62.4
2′	103.6	104.0	104.0
3′	77.4	77.1	77.1
4′	74.4	74.8	78.7
5′	81.0	81.5	81.5
6′	60.8	60.7	60.7
1″	60.3	60.5	60.5
2″	103.6	104.0	104.0
3″	76.7	77.7	77.7
4″	74.3	74.8	74.8
5″	81.0	81.5	81.5
6″	62.2	62.5	62.5
1‴	60.0	65.9	65.9
2‴	103.1	104.0	104.0
3‴	76.6	76.9	76.9
4‴	73.7	74.6	74.6
5‴	81.0	81.3	81.3
6‴	62.3	62.5	62.5
buty-1		176.9	176.9
buty-2		36.0	36.0
buty-3		18.3	18.3
buty-4		13.2	13.2
buty-1′			176.2
buty-2′			36.0
buty-3′			18.2
buty-4′			13.1

**Table 3:** Changes in short-chain fatty acid (SCFA) production and pH by 24 h anaerobic batch fermentation of fecal samples. Data are expressed as mean ± standard deviation (SD) mM (*n* = 3).

Groups	Changes of SCFA Concentrations (mM)			pH
	Acetate	Propionate	Butyrate	
Control	19.33 ± 0.82 ^c^	4.15 ± 0.12	9.42 ± 0.28 ^b^	6.46 ± 0.03 ^a^
FOS	26.09 ± 0.67 ^a^	3.91 ± 0.42	3.86 ± 0.07 ^c^	5.45 ± 0.02 ^b^
B-FOS	21.07 ± 0.64 ^b^	4.04 ± 0.73	18.84 ± 1.05 ^a^	5.68 ± 0.02 ^ab^

Treatments with different letters are significantly different at *p* < 0.05.

**Table 4:** Relative abundance of the selected butyrate-producing bacteria (n = 3)**.**

Genus	Relative Abundance (%)			B-FOS
	Before	Control	FOS	
*Faecalibacterium*	3.36 ± 0.14 ^a^	0.19 ± 0.06 ^b^	0.59 ± 0.05 ^ab^	2.67 ± 0.38 ^ab^
*Ruminococcus*	2.25 ± 0.16 ^a^	0.19 ± 0.03 ^b^	0.34 ± 0.10 ^ab^	1.08 ± 0.12 ^ab^
*Clostridium*	0.21 ± 0.04 ^ab^	0.38 ± 0.07 ^ab^	0.18 ± 0.07 ^b^	1.16 ± 0.06 ^a^
*Oscillospira*	4.37 ± 0.07 ^a^	0.30 ± 0.09 ^b^	0.71 ± 0.08 ^ab^	0.85 ± 0.12 ^ab^
*Collinsella*	0.43 ± 0.03 ^ab^	0.20 ± 0.01 ^b^	0.31 ± 0.10 ^ab^	0.91 ± 0.18 ^a^

Treatments with different letters are significantly different at *p* < 0.05.

## Supplementary Materials

The following are available online at https://www.mdpi.com/1422-0067/21/2/445/s1: Figure S1. Determination of butyl-fructooligosaccharides during purification by TLC, Figure S2. ^1^H NMR spectra and ^13^C NMR spectra of B-FOS, Figure S3. Chemical structures of fructooligosaccharides 1–3 and fragmentations by negative low-resolution fast-atom bombardment mass spectra (LR-FAB-MS), Figure S4. Relative abundances of butanoate metabolism-associated Kyoto Encyclopedia of Genes and Genomes (KEGG) genes that were significantly abundant in the B-FOS treatment amongst the groups, Figure S5. Relative abundances of butanoate metabolism associated KEGG genes that were significantly abundant in the control amongst the groups, Figure S6. Relative abundances of butanoate metabolism associated KEGG genes that were significantly abundant in the FOS treatment amongst the groups, Figure S7. Correlations between butyrate production and relative abundances of butanoate metabolism-related KEGG genes that were significantly abundant in the B-FOS treatment amongst the groups, Figure S8. KEGG analysis associated with ammonia production pathways.

## References

[1] Hamer H.M., Jonkers D., Venema K., Vanhoutvin S., Troost F., Brummer R.J. (2008). The role of butyrate on colonic function. Aliment. Pharmacol. Ther..

[2] Gonçalves P., Araújo J.R., Martel F. (2011). Characterization of butyrate uptake by nontransformed intestinal epithelial cell lines. J. Membr. Biol..

[3] Van Immerseel F., Ducatelle R., De Vos M., Boon N., Van De Wiele T., Verbeke K., Rutgeerts P., Sas B., Louis P., Flint H.J. (2010). Butyric acid-producing anaerobic bacteria as a novel probiotic treatment approach for inflammatory bowel disease. J. Med. Microbiol..

[4] Thibault R., Blachier F., Darcy-Vrillon B., De Coppet P., Bourreille A., Segain J.-P. (2009). Butyrate utilization by the colonic mucosa in inflammatory bowel diseases: A transport deficiency. Inflamm. Bowel Dis..

[5] Leonel A.J., Alvarez-Leite J.I. (2012). Butyrate: Implications for intestinal function. Curr. Opin. Clin. Nutr. Metab. Care.

[6] Simeoli R., Mattace Raso G., Pirozzi C., Lama A., Santoro A., Russo R., Montero-Melendez T., Berni Canani R., Calignano A., Perretti M. (2017). An orally administered butyrate-releasing derivative reduces neutrophil recruitment and inflammation in dextran sulphate sodium-induced murine colitis. Br. J. Pharmacol..

[7] Fu X., Liu Z., Zhu C., Mou H., Kong Q. (2018). Nondigestible carbohydrates, butyrate, and butyrate-producing bacteria. Crit. Rev. Food Sci. Nutr..

[8] Tuleu C., Andrieux C., Cherbuy C., Darcy-Vrillon B., Duee P., Chaumeil J. (2001). Colonic delivery of sodium butyrate via oral route: Acrylic coating design of pellets and in vivo evaluation in rats. Methods Find. Exp. Clin. Pharmacol..

[9] Roda A., Simoni P., Magliulo M., Nanni P., Baraldini M., Roda G., Roda E. (2007). A new oral formulation for the release of sodium butyrate in the ileo-cecal region and colon. World J. Gastroenterol..

[10] Bajka B.H., Clarke J.M., Cobiac L., Topping D.L. (2008). Butyrylated starch protects colonocyte DNA against dietary protein-induced damage in rats. Carcinogenesis.

[11] Srinivas S.R., Prasad P.D., Umapathy N.S., Ganapathy V., Shekhawat P.S. (2007). Transport of butyryl-l-carnitine, a potential prodrug, via the carnitine transporter OCTN2 and the amino acid transporter ATB^0,+^. Am. J. Physiol. Gastr. Liver Physiol..

[12] Frank D.N., Amand A.L.S., Feldman R.A., Boedeker E.C., Harpaz N., Pace N.R. (2007). Molecular-phylogenetic characterization of microbial community imbalances in human inflammatory bowel diseases. Proc. Natl. Acad. Sci. USA.

[13] Mussatto S.I., Mancilha I.M. (2007). Non-digestible oligosaccharides: A review. Carbohydr. Polym..

[14] Yun J.W. (1996). Fructooligosaccharides—Occurrence, preparation, and application. Enzyme Microb. Technol..

[15] Sabater-Molina M., Larqué E., Torrella F., Zamora S. (2009). Dietary fructooligosaccharides and potential benefits on health. J. Physiol. Biochem..

[16] Mao B., Li D., Zhao J., Liu X., Gu Z., Chen Y.Q., Zhang H., Chen W. (2015). Metagenomic insights into the effects of fructo-oligosaccharides (FOS) on the composition of fecal microbiota in mice. J. Agric. Food Chem..

[17] Shen J., Zhang B., Wei H., Che C., Ding D., Hua X., Bucheli P., Wang L., Li Y., Pang X. (2010). Assessment of the modulating effects of fructo-oligosaccharides on fecal microbiota using human flora–associated piglets. Arch. Microbiol..

[18] Van Laere K.M., Hartemink R., Bosveld M., Schols H.A., Voragen A.G. (2000). Fermentation of plant cell wall derived polysaccharides and their corresponding oligosaccharides by intestinal bacteria. J. Agric. Food Chem..

[19] Patel S., Goyal A. (2012). The current trends and future perspectives of prebiotics research: A review. 3 Biotech.

[20] Bui N.Q., Fongarland P., Rataboul F., Dartiguelongue C., Charon N., Vallée C., Essayem N. (2015). FTIR as a simple tool to quantify unconverted lignin from chars in biomass liquefaction process: Application to SC ethanol liquefaction of pine wood. Fuel Process. Technol..

[21] Proniewicz L.M., Paluszkiewicz C., Wesełucha-Birczyńska A., Majcherczyk H., Barański A., Konieczna A. (2001). FT-IR and FT-Raman study of hydrothermally degradated cellulose. J. Mol. Struct..

[22] Synytsya A., Novak M. (2014). Structural analysis of glucans. Ann. Transl. Med..

[23] Hung W.-T., Wang S.-H., Chen Y.-T., Yu H.-M., Chen C.-H., Yang W.-B. (2012). MALDI-TOF MS analysis of native and permethylated or benzimidazole-derivatized polysaccharides. Molecules.

[24] Cerantola S., Kervarec N., Pichon R., Magné C., Bessieres M.-A., Deslandes E. (2004). NMR characterisation of inulin-type fructooligosaccharides as the major water-soluble carbohydrates from *Matricaria maritima* (L.). Carbohyd. Res..

[25] Santos-Moriano P., Fernandez-Arrojo L., Poveda A., Jimenez-Barbero J., Ballesteros A.O., Plou F.J. (2015). Levan versus fructooligosaccharide synthesis using the levansucrase from Zymomonas mobilis: Effect of reaction conditions. J. Mol. Catal. B Enzym..

[26] Jiang H., Ling Z., Zhang Y., Mao H., Ma Z., Yin Y., Wang W., Tang W., Tan Z., Shi J. (2015). Altered fecal microbiota composition in patients with major depressive disorder. Brain Behav. Immun..

[27] Louis P., Flint H.J. (2009). Diversity, metabolism and microbial ecology of butyrate-producing bacteria from the human large intestine. FEMS Microbiol. Lett..

[28] Hold G.L., Schwiertz A., Aminov R.I., Blaut M., Flint H.J. (2003). Oligonucleotide probes that detect quantitatively significant groups of butyrate-producing bacteria in human feces. Appl. Environ. Microbiol..

[29] Barcenilla A., Pryde S.E., Martin J.C., Duncan S.H., Stewart C.S., Henderson C., Flint H.J. (2000). Phylogenetic relationships of butyrate-producing bacteria from the human gut. Appl. Environ. Microbiol..

[30] Gophna U., Konikoff T., Nielsen H.B. (2017). Oscillospira and related bacteria–From metagenomic species to metabolic features. Environ. Microbiol..

[31] Qin P., Zou Y., Dai Y., Luo G., Zhang X., Xiao L. (2019). Characterization a Novel Butyric Acid-Producing Bacterium Collinsella aerofaciens Subsp. Shenzhenensis Subsp. Nov. Microorganisms.

[32] Meletis C.D., Zabriskie N. (2008). Supporting gastrointestinal health with nutritional therapy. J. Altern. Complement. Med..

[33] Nowak A., Libudzisz Z. (2006). Influence of phenol, p-cresol and indole on growth and survival of intestinal lactic acid bacteria. Anaerobe.

[34] Rycroft C., Jones M., Gibson G.R., Rastall R. (2001). Fermentation properties of gentio-oligosaccharides. Lett. Appl. Microbiol..

[35] Fehlbaum S., Prudence K., Kieboom J., Heerikhuisen M., van den Broek T., Schuren F., Steinert R., Raederstorff D. (2018). In vitro fermentation of selected prebiotics and their effects on the composition and activity of the adult gut microbiota. Int. J. Mol. Sci..

[36] Gu J., Mao B., Cui S., Liu X., Zhang H., Zhao J., Chen W. (2019). Metagenomic Insights into the Effects of Fructooligosaccharides (FOS) on the Composition of Luminal and Mucosal Microbiota in C57BL/6J Mice, Especially the Bifidobacterium Composition. Nutrients.

[37] Wu T.-R., Lin C.-S., Chang C.-J., Lin T.-L., Martel J., Ko Y.-F., Ojcius D.M., Lu C.-C., Young J.D., Lai H.-C. (2019). Gut commensal Parabacteroides goldsteinii plays a predominant role in the anti-obesity effects of polysaccharides isolated from Hirsutella sinensis. Gut.

[38] Martínez I., Kim J., Duffy P.R., Schlegel V.L., Walter J. (2010). Resistant starches types 2 and 4 have differential effects on the composition of the fecal microbiota in human subjects. PLoS ONE.

[39] Haro C., Montes-Borrego M., Rangel-Zúñiga O.A., Alcalá-Díaz J.F., Gómez-Delgado F., Pérez-Martínez P., Delgado-Lista J., Quintana-Navarro G.M., Tinahones F.J., Landa B.B. (2016). Two healthy diets modulate gut microbial community improving insulin sensitivity in a human obese population. J. Clin. Endocrinol. Metab..

[40] Wang K., Liao M., Zhou N., Bao L., Ma K., Zheng Z., Wang Y., Liu C., Wang W., Wang J. (2019). Parabacteroides distasonis alleviates obesity and metabolic dysfunctions via production of succinate and secondary bile acids. Cell Rep..

[41] Barengolts E., Green S.J., Chlipala G.E., Layden B.T., Eisenberg Y., Priyadarshini M., Dugas L.R. (2019). Predictors of Obesity among Gut Microbiota Biomarkers in African American Men with and without Diabetes. Microorganisms.

[42] Konikoff T., Gophna U. (2016). Oscillospira: A central, enigmatic component of the human gut microbiota. Trends Microbiol..

[43] Kelly T.N., Bazzano L.A., Ajami N.J., He H., Zhao J., Petrosino J.F., Correa A., He J. (2016). Gut microbiome associates with lifetime cardiovascular disease risk profile among bogalusa heart study participants. Circ. Res..

[44] Greenwood D., Slack R.C., Barer M.R., Irving W.L. (2012). Medical Microbiology E-Book: A Guide to Microbial Infections: Pathogenesis, Immunity, Laboratory Diagnosis and Control.

[45] Liu F., Li P., Chen M., Luo Y., Prabhakar M., Zheng H., He Y., Qi Q., Long H., Zhang Y. (2017). Fructooligosaccharide (FOS) and galactooligosaccharide (GOS) increase *Bifidobacterium* but reduce butyrate producing bacteria with adverse glycemic metabolism in healthy young population. Sci. Rep..

[46] Ashley D., Marasini D., Brownmiller C., Lee J., Carbonero F., Lee S.-O. (2019). Impact of grain sorghum polyphenols on microbiota of normal weight and overweight/obese subjects during in vitro fecal fermentation. Nutrients.

[47] Xie Z., Wang S., Wang Z., Fu X., Huang Q., Yuan Y., Wang K., Zhang B. (2019). In vitro *f*ecal fermentation of propionylated high-amylose maize starch and its impact on gut microbiota. Carbohyd. Polym..

[48] Tochio T., Kadota Y., Tanaka T., Koga Y. (2018). 1-Kestose, the smallest fructooligosaccharide component, which efficiently stimulates *Faecalibacterium prausnitzii* as well as bifidobacteria in humans. Foods.

[49] Sokol H., Pigneur B., Watterlot L., Lakhdari O., Bermúdez-Humarán L.G., Gratadoux J.-J., Blugeon S., Bridonneau C., Furet J.-P., Corthier G. (2008). *Faecalibacterium prausnitzii* is an anti-inflammatory commensal bacterium identified by gut microbiota analysis of Crohn disease patients. Proc. Natl. Acad. Sci. USA.

[50] Quévrain E., Maubert M., Michon C., Chain F., Marquant R., Tailhades J., Miquel S., Carlier L., Bermúdez-Humarán L., Pigneur B. (2016). Identification of an anti-inflammatory protein from *Faecalibacterium prausnitzii*, a commensal bacterium deficient in Crohn’s disease. Gut.

[51] Saalman R., Alderberth I., Wold A., Sjoberg F. (2018). Use of Collinsella for Treatment of Inflammatory Bowel Disease. U.S. Patent.

[52] Anand S., Kaur H., Mande S.S. (2016). Comparative in silico analysis of butyrate production pathways in gut commensals and pathogens. Front. Microbiol..

[53] Aliyu S., Marriott R., Curran M., Parmar S., Bentley N., Brown N., Brazier J., Ludlam H. (2004). Real-time PCR investigation into the importance of *Fusobacterium necrophorum* as a cause of acute pharyngitis in general practice. J. Med. Microbiol..

[54] Biedrzycka E., Bielecka M. (2004). Prebiotic effectiveness of fructans of different degrees of polymerization. Trends Food Sci. Technol..

[55] Pinna C., Vecchiato C.G., Zaghini G., Grandi M., Nannoni E., Stefanelli C., Biagi G. (2016). In vitro influence of dietary protein and fructooligosaccharides on metabolism of canine fecal microbiota. BMC Vet. Res..

[56] Louis P., Flint H.J. (2017). Formation of propionate and butyrate by the human colonic microbiota. Environ. Microbiol..

[57] Vital M., Howe A.C., Tiedje J.M. (2014). Revealing the bacterial butyrate synthesis pathways by analyzing (meta) genomic data. MBio.

[58] Strauss J., Kaplan G.G., Beck P.L., Rioux K., Panaccione R., DeVinney R., Lynch T., Allen-Vercoe E. (2011). Invasive potential of gut mucosa-derived *Fusobacterium nucleatum* positively correlates with IBD status of the host. Inflamm. Bowel Dis..

[59] McCoy A.N., Araujo-Perez F., Azcarate-Peril A., Yeh J.J., Sandler R.S., Keku T.O. (2013). *Fusobacterium* is associated with colorectal adenomas. PLoS ONE.

[60] Wong J.M., De Souza R., Kendall C.W., Emam A., Jenkins D.J. (2006). Colonic health: Fermentation and short chain fatty acids. J. Clin. Gastroenterol..

[61] Tsitko I., Wiik-Miettinen F., Mattila O., Rosa-Sibakov N., Seppänen-Laakso T., Maukonen J., Nordlund E., Saarela M. (2019). A small in vitro fermentation model for screening the gut microbiota effects of different fiber preparations. Int. J. Mol. Sci..

[62] Rycroft C., Jones M., Gibson G.R., Rastall R. (2001). A comparative in vitro evaluation of the fermentation properties of prebiotic oligosaccharides. J. Appl. Microbiol..

[63] Min B., Koo O.K., Park S.H., Jarvis N., Ricke S.C., Crandall P.G., Lee S.-O. (2015). Fermentation patterns of various pectin sources by human fecal microbiota. Food Nutr. Sci..

[64] Tejada-Ortigoza V., Garcia-Amezquita L.E., Kazem A.E., Campanella O.H., Cano M.P., Hamaker B.R., Serna-Saldívar S.O., Welti-Chanes J. (2019). In vitro fecal fermentation of high pressure-treated fruit peels used as dietary fiber sources. Molecules.

[65] Beards E., Tuohy K., Gibson G. (2010). Bacterial, SCFA and gas profiles of a range of food ingredients following in vitro fermentation by human colonic microbiota. Anaerobe.

[66] Chaney A.L., Marbach E.P. (1962). Modified reagents for determination of urea and ammonia. Clin. Chem..

