# Supplementary material for: Production, Structural Characterization, and In Vitro Assessment of the Prebiotic Potential of Butyl-Fructooligosaccharides
Source: Int J Mol Sci. 2020 Jan 10;21(2):445. doi: 10.3390/ijms21020445 (PMC7013684; doi:10.3390/ijms21020445)
Supplement: Supplementary file 1 [file ijms-21-00445-s001.pdf]

Supplementary Materials: Figure S1. Determination of butyl-fructooligosaccharides during purification by TLC, Figure S2. ^1H NMR spectra and ^{13}C NMR spectra of B-FOS, Figure S3. Chemical structures of fructooligosaccharides 1-3 and fragmentations by negative LR-FAB-MS spectra, Figure S4. Relative abundances of butanoate metabolism-associated KEGG genes that were significantly abundant in the B-FOS treatment amongst the groups, Figure S5. Relative abundances of butanoate metabolism associated KEGG genes that were significantly abundant in the control amongst the groups, Figure S6. Relative abundances of butanoate metabolism associated KEGG genes that were significantly abundant in the FOS treatment amongst the groups, Figure 7. Correlations between butyrate production and relative abundances of butanoate metabolism-related KEGG genes that were significantly abundant in the B-FOS treatment amongst the groups, Figure 8. KEGG analysis associated with ammonia production pathways.

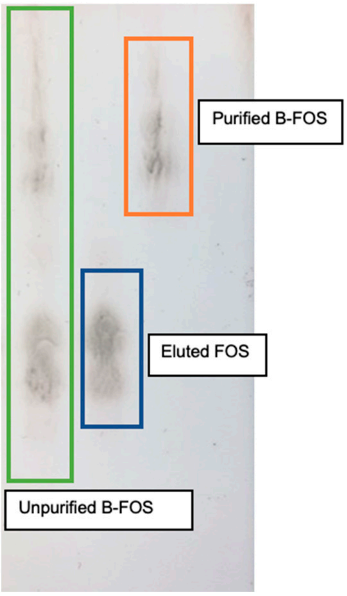

Figure S1. Determination of butyl-fructooligosaccharides during purification by TLC.

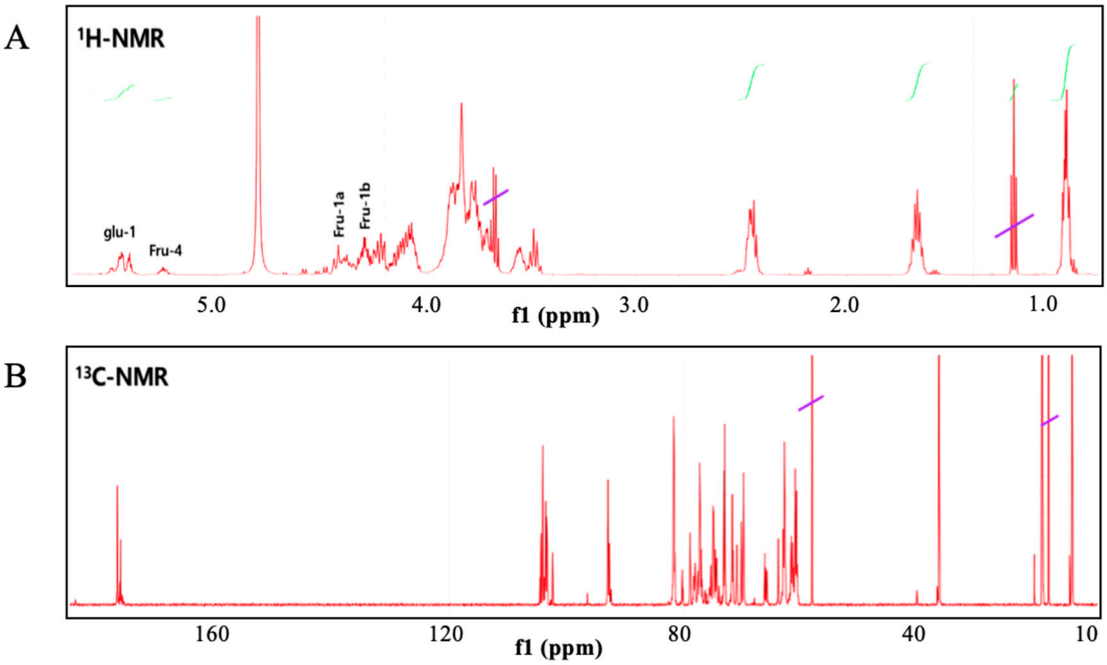

Figure S2. ^{1}H NMR spectra and ^{13}C NMR spectra of B-FOS. The peaks removed by violet lines were EtOH remaining during purification.

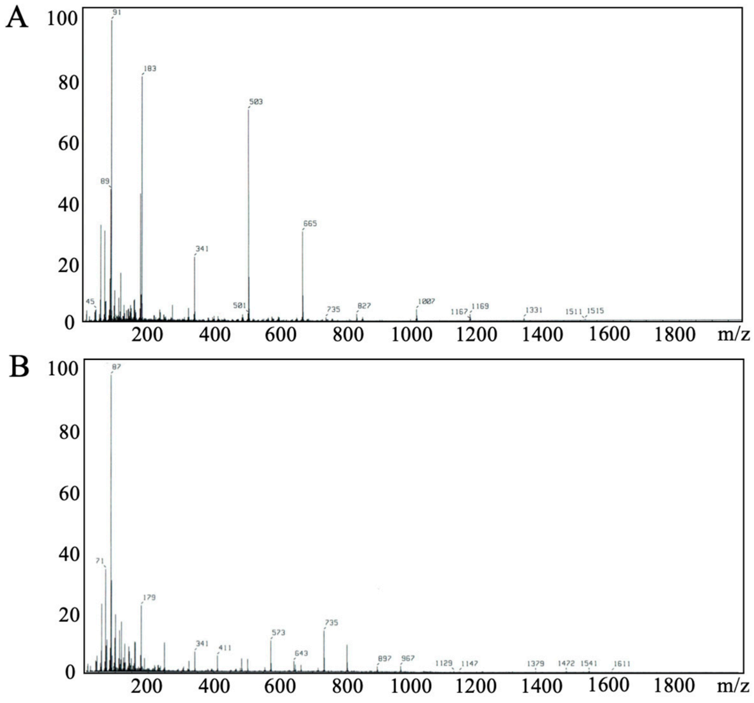

Figure S3. Fragmentations by negative LR-FAB-MS spectra of (A) FOS and (B) B-FOS.

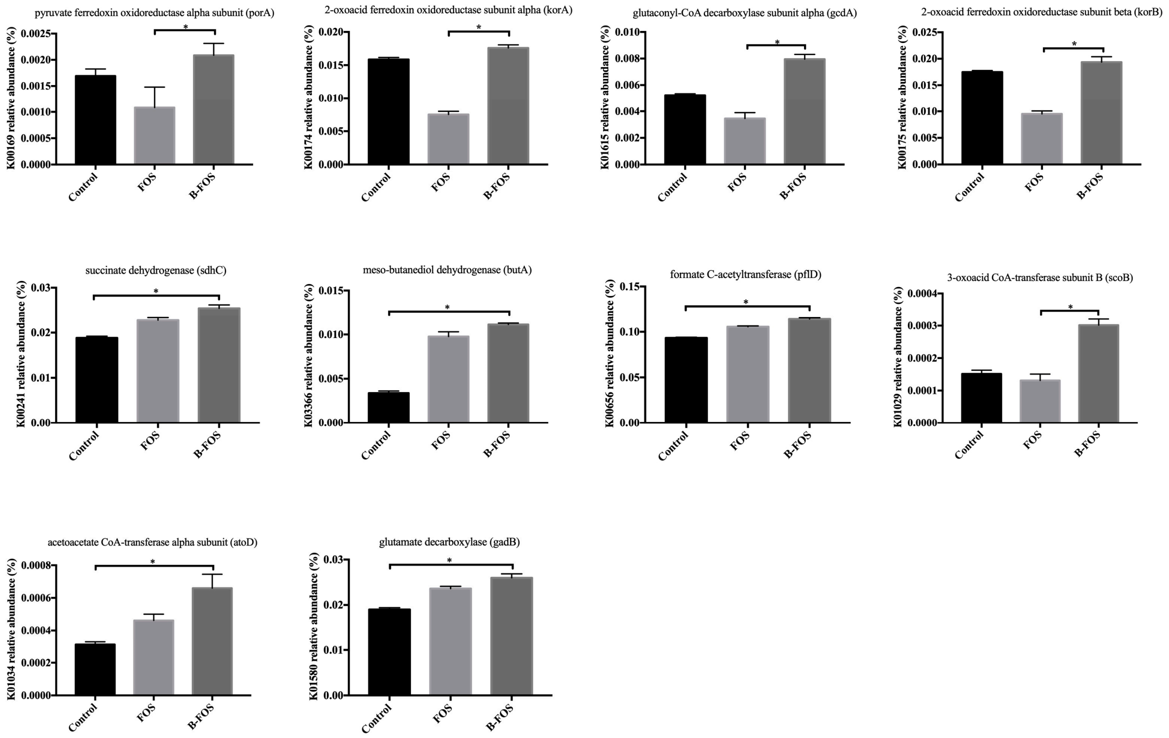

Figure S4. Relative abundances of butanoate metabolism-associated KEGG genes that were significantly abundant in the B-FOS treatment amongst groups. Significance was accepted at $p < 0.05$.

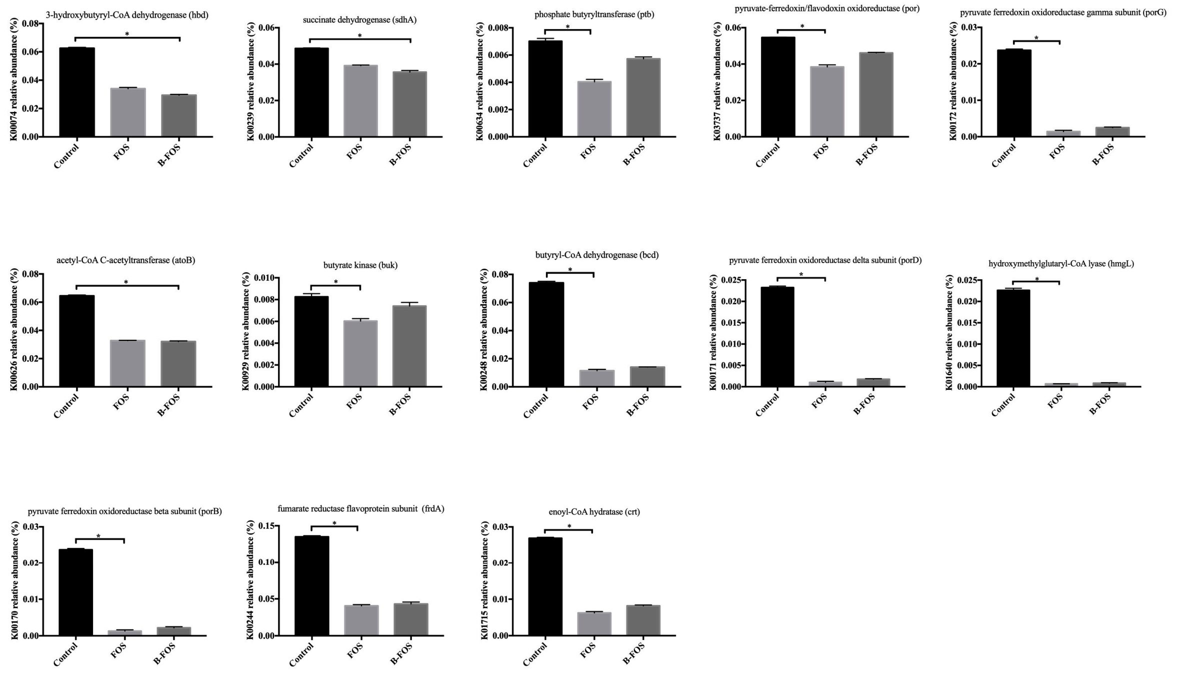

Figure S5. Relative abundances of butanoate metabolism associated KEGG genes that were significantly abundant in the control amongst groups. Significance was accepted at $p < 0.05$.

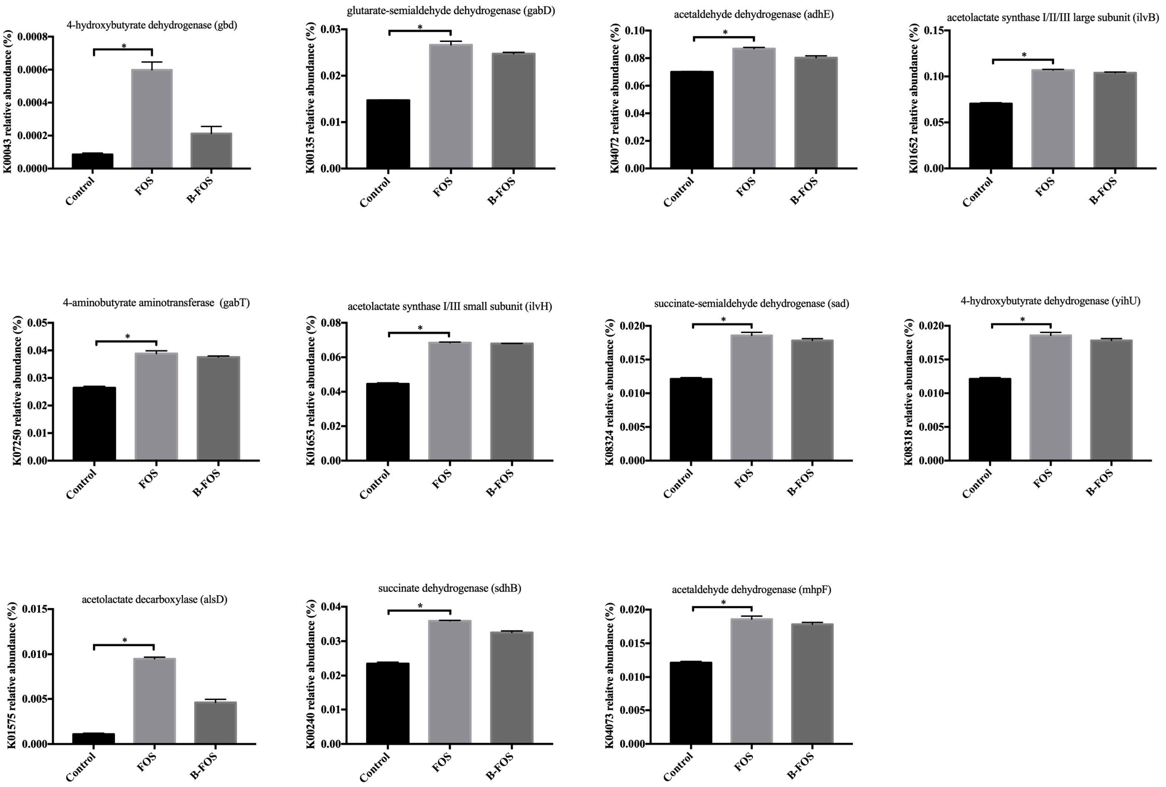

Figure S6. Relative abundances of butanoate metabolism associated KEGG genes that were significantly abundant in the FOS treatment amongst groups. Significance was accepted at $p < 0.05$.

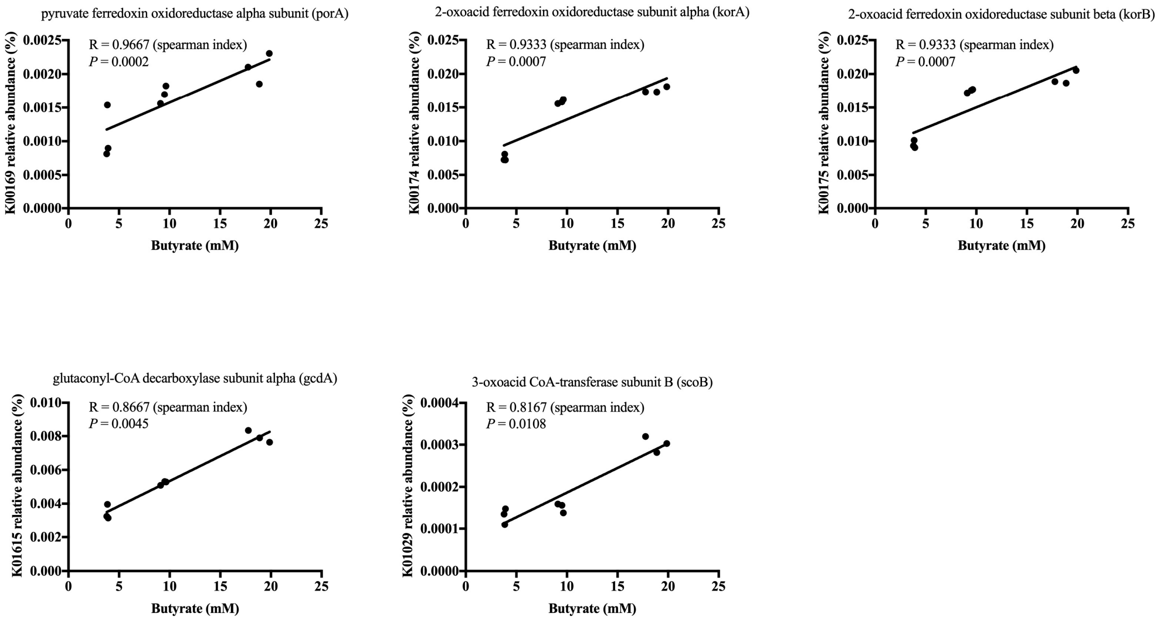

Figure S7. Correlations between butyrate production and relative abundances of butanoate metabolism-related KEGG genes that were significantly abundant in the B-FOS treatment amongst groups. Significance was accepted at $p < 0.05$.

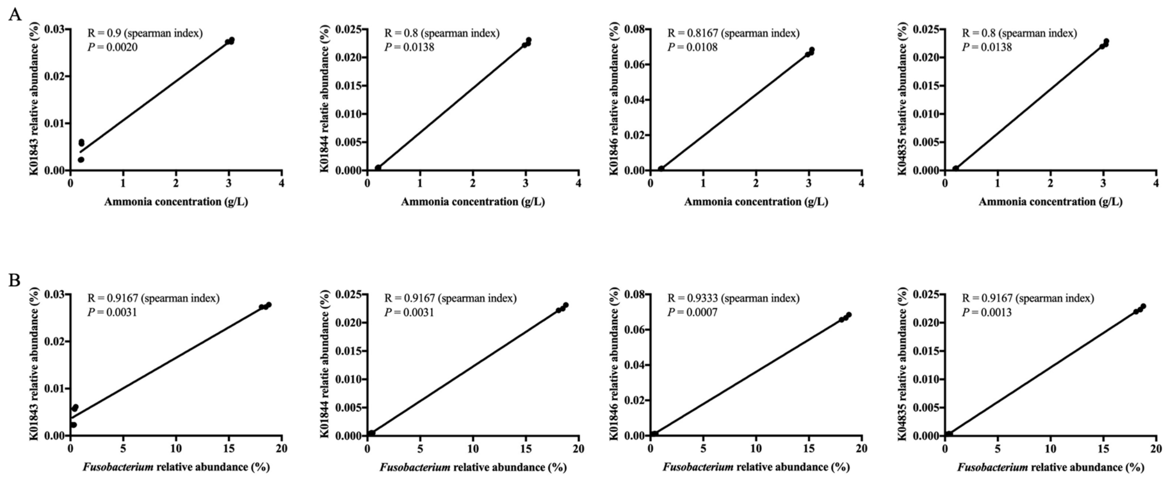

Figure S8. KEGG analysis associated with ammonia production pathways. (A) correlations between ammonia concentrations and ammonia production-related KEGG genes (B) correlations between relative abundance of *Fusobacteirum* and ammonia production-related KEGG genes. Significance was accepted at $p < 0.05$.
